# Physiological Outperformance at the Morphologically-Transformed Edge of the Cyanobacteriosponge *Terpios hoshinota* (Suberitidae: Hadromerida) when Confronting Opponent Corals

**DOI:** 10.1371/journal.pone.0131509

**Published:** 2015-06-25

**Authors:** Jih-Terng Wang, Chia-Min Hsu, Chao-Yang Kuo, Pei-Jie Meng, Shuh-Ji Kao, Chaolun Allen Chen

**Affiliations:** 1 Graduate Institute of Biotechnology, Tajen University, Pingtung, Taiwan; 2 Institute of Oceanography, National Taiwan University, Taipei, Taiwan; 3 Biodiversity Research Center, Academia Sinica, Taipei, Taiwan; 4 ARC Centre for Coral Reef Studies, James Cook University, Townsville, Australia; 5 National Museum of Marine Biology and Aquarium, Checheng, Pingtung, Taiwan; 6 Institute of Marine Biodiversity and Evolution, National Dong Hwa University Checheng, Pingtung, Taiwan; 7 State Key Laboratory of Marine Environmental Science, Xiamen University, Xiamen, China; University of Genova, Italy, ITALY

## Abstract

*Terpios hoshinota*, an encrusting cyanosponge, is known as a strong substrate competitor of reef-building corals that kills encountered coral by overgrowth. *Terpios* outbreaks cause significant declines in living coral cover in Indo-Pacific coral reefs, with the damage usually lasting for decades. Recent studies show that there are morphological transformations at a sponge’s growth front when confronting corals. Whether these morphological transformations at coral contacts are involved with physiological outperformance (*e*.*g*., higher metabolic activity or nutritional status) over other portions of *Terpios* remains equivocal. In this study, we compared the indicators of photosynthetic capability and nitrogen status of a sponge-cyanobacteria association at proximal, middle, and distal portions of opponent corals. *Terpios* tissues in contact with corals displayed significant increases in photosynthetic oxygen production (*ca*. 61%), the δ^13^C value (*ca*. 4%), free proteinogenic amino acid content (*ca*. 85%), and Gln/Glu ratio (*ca*. 115%) compared to middle and distal parts of the sponge. In contrast, the maximum quantum yield (*F_v_/F_m_*), which is the indicator usually used to represent the integrity of photosystem II, of cyanobacteria photosynthesis was low (0.256~0.319) and showed an inverse trend of higher values in the distal portion of the sponge that might be due to high and variable levels of cyanobacterial phycocyanin. The inconsistent results between photosynthetic oxygen production and *F_v_/F_m_* values indicated that maximum quantum yields might not be a suitable indicator to represent the photosynthetic function of the *Terpios*-cyanobacteria association. Our data conclusively suggest that *Terpios hoshinota* competes with opponent corals not only by the morphological transformation of the sponge-cyanobacteria association but also by physiological outperformance in accumulating resources for the battle.

## Introduction

Competition among sessile organisms is one of the major ecological processes of coral reefs and can result in altering coral species diversity and abundance at spatial and temporal scales (reviewed in [1,2]). As the impacts of climate change and human disturbances increase, including anomalous seawater temperatures, overfishing of herbivores, increased levels of dissolved nutrients, sedimentation, and physical disturbances (*e*.*g*., typhoons), the competitive capacity of coral opponents like algae, sponges, ascidians, and skeleton-less cnidarians can be enhanced if disturbances favor the growth of sessile organisms. As a consequence, large-scale phase shifts in reefs from coral dominance to alternative steady states dominated by algae or sponges is occurring in tropical coral reefs [3,4,5].

**Fig 1:**
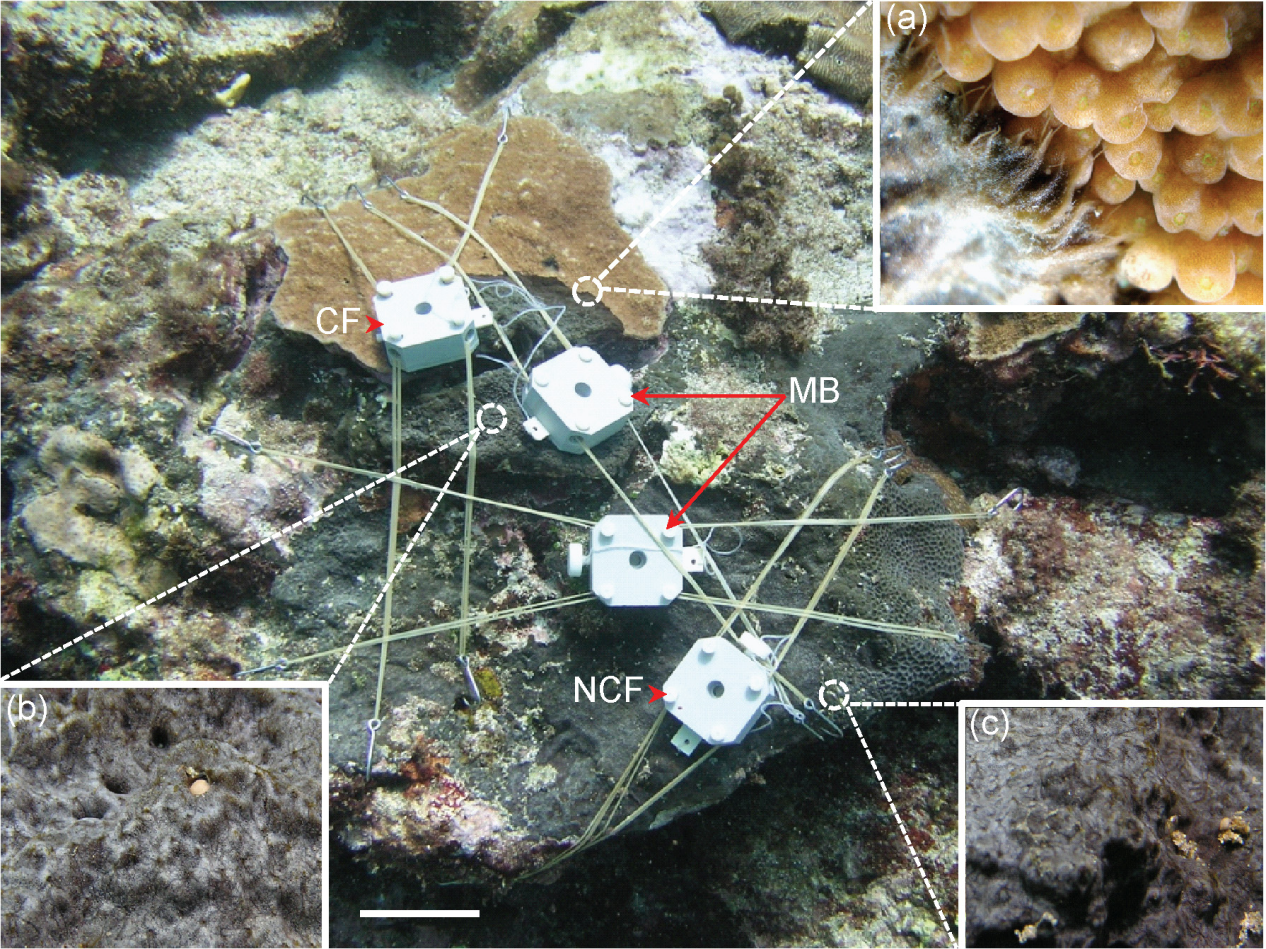
Sampling sites on a *Terpios hoshinota* colony used for field determination of maximum quantum yield and other laboratory analyses after excising living coral skeletons with imbedded sponge tissues at the coral front (CF), mid-body (MB), and no-contact coral front (NCF). The white box affixed by elastic strings is the probe holder for the DIVING-PAM fluorometer. Insets are close-up photos of the sponge at (a) CF, (b) MB, and (c) NCF. The scale bar on the bottom is 10 cm.

Cyanosponges are fast growing and aggressively competitive in disturbed areas, overgrowing and killing live corals [6–8, 9]. In the Indo-West Pacific, *Terpios hoshinota* is reported as a cyanosponge species that outcompetes scleractinian corals and causes significant declines in living coral cover during outbreaks over the last four decades [7,10–18]. *Terpios* is a very thin (<1 mm) and black sheet-like sponge that firmly encrusts corals, with sponge tissue penetrating deeply into the steeply undulating coral skeleton [19]. The surface of the sponge is usually free from epibionts and sometimes contains calcareous grains underneath [19]. *Terpios* can be categorized as a “phototrophic” cyanosponge based on its flattened morphology [20,21] and the presence of extremely dense populations of photosynthetic cyanobacteria in the mesohyl [7, 9–12]. Histological examinations indicate that endosymbiotic cyanobacteria in the *Terpios* mesohyl occupy > 50% of total cell volume and may contribute to *Terpios* coloration [7,19]. Molecular phylogenetic analysis using the 16 rRNA gene demonstrates that the dominant cyanobacteria taxon is an isolated group that is closely related to *Prochloron* spp. [22], suggesting that this strain of cyanobacteria also contains phycocyanin [19] and probably chlorophyll *b* [9]. *Terpios* prefers to grow on living coral rather than other substrates [13]. When encountering coral opponents, *Terpios* develops four different types of morphological reactions [23]. “Hairy” tips packed with cyanobacteria, sponge tissues, and spicules were the most frequently observed reaction of sponges to corals (Fig 1). *Terpios* can also transform from an encrusting sheet-like structure into a thread-like tissue in order to move across a shaded area or reach new territory [23,24]. Occasionally, *Terpios* exhibits negative growth and can even be overgrown by certain coral species or red calcareous algae [11,23].

Although diverse morphological responses were observed at the contact between *Terpios hoshinota* and opponent corals, the physiological performance of the sponge-cyanobacteria association in this interaction remains unknown. As cyanobacteria occupy a significant proportion of the biomass within sponges [22], measuring the physiological performance of cyanobacteria may represent the overall status of *Terpios*. In this study, we examined maximum quantum yield, photosynthetic oxygen production, and chlorophyll contents to represent photosynthetic performance, free amino acid content to represent nitrogen status, andδ^13^C andδ^15^N stable isotope ratios to represent metabolic activity in the sponge-cyanobacteria association at proximal, middle, and distal regions of opponent corals. These physiological and metabolic indices measured from different parts of the sponge suggest that *Terpios hoshinota* exhibits physiological outperformance where it contacts coral and that this might benefit the sponge-cyanobacteria association in accumulating the resources to compete with coral.

## Materials and Methods

### Ethics statement

No specific permissions were required for the collection of *Terpios* specimens at Gong-guan, Green Island (22°39’N, 121°29’E)”, according to local government regulations. We also confirmed that our field studies did not involve endangered or protected species.

### Study site, field experiments, and sample collection

Field experiments and *T*. *hoshinota* sample collections were conducted in May, July, and November, 2010, at Gong-guan, Green Island (22°39’N, 121°29’E), in 2007 where Taiwan’s first outbreak of *Terpios* was reported [12]. Colonies of *T*. *hoshinota* (approximately 50 cm in diameter) attacking catch bowl coral, *Isopora palifera*, and showing “hairy” tip structures during attacks on corals (Fig 1) were selected for *in situ* photosynthetic measurements and follow-on physiological and metabolic examination in the laboratory. Different parts of *Terpios* colonies (the growth front contacting the living coral “CF“, mid-body “MB“, and no-contact front “NCF“), were selected for photosynthetic measurements and collections for laboratory experiments (Fig 1).

### Maximum quantum yield determination *in situ*

In order to evaluate the *in situ* photosynthetic functions of *Terpios* cyanobacteria, maximum quantum yields of PSII (*F*_*v*_ */F*_*m*_ = (*F*_*m*_—*F*_*o*_)/ *F*_*m*_) were determined using a DIVING-PAM fluorometer (Walz, Germany) following the method developed for coral and photosynthetic sponge studies [25–27]. Data for *Terpios* MB were pooled from two measurements taken from each sponge colony. Nontransparent, custom-made DIVING-PAM probe holders were affixed to *Terpios* CF, MB, and NCF for 30 min dark adaption (Fig 1), after which minimum fluorescence (*F*_*o*_) was measured at a measuring-light setting of 8. This was followed immediately by a saturating flash of actinic light (DIVING-PAM setting 8) and the measurement of maximum fluorescence (*F*_*m*_).

### Laboratory measurement of photosynthetic capability and dark respiration rate

Representative samples of *Terpios* approximately 5 cm in diameter for Diving-PAM measurements were chiseled from sponges to determine photosynthetic oxygen evolution and respiration rates. Each sample was sealed in an individual plastic bag underwater for delivery to the maintenance tank to avoid desiccation. *Terpios* samples were temporarily maintained in an aerated tank (180 × 60 × 30 cm) under low light (< 20 μmol photons m^-2^ s^-1^) with two underwater pumps (1800 L h^-1^) to create strong water flow. Sponge samples were used for experiments or isolation of cyanobacteria within a day of collection.

Oxygen flux due to *Terpios* photosynthesis and respiration symbiosis was measured in a custom-made respiration chamber (400 ml) with a BOD probe (YSI 5905, Yellow Springs, OH, USA) and dissolved oxygen (DO) meter (YSI 52). Oxygen concentration changes in the respiration chamber were continuously measured for 15 min by connecting the meters to a personal computer using manufacturer software. The *Terpios* symbiosis dark respiration rate was measured by covering the respiration chamber with a black sheet. *Terpios* symbiosis photosynthesis was induced with a UV-block halogen light bulb. Photosynthesis irradiance was checked with a quantum photometer (LI-COR, model LI-250 Light Meter, Lincoln, NE, USA) attached to a 2π sensor (LI-COR, model: QUANTUM) and expressed as μmol photons m^-2^s^-1^ P.A.R. Light intensity was increased to determine the photosynthesis-irradiation curve by moving the light source closer to the respiration chamber, which did not result in elevating the temperature of the chamber during the experiment. After determining the changes in oxygen concentration, respiration and photosynthesis rates were standardized to the area of the dried coral substrate using the aluminum foil method [28].

### Isolation of cyanobacteria and physiological index determination

Live tissue from each *Terpios* sample (*ca*. 25 cm^2^) was first scraped with a spatula and stripped of remaining tissue with a pressurized air-seawater blast, modified from a method used for coral [29]. Artificial seawater (ASW) for air blasts was prepared from sea salt (Instant Ocean, Aquarium Systems, Sarrebourg Cedex, France) and pre-cooled on ice. The tissue slurry (*ca*. 10 ml) was then homogenized in a hand-held tissue grinder in an ice bath. Tissues were homogenized and left to stand for 3 min to precipitate heavy debris such as coral skeleton particles and spicules, and the resulting supernatant was filtered through 15 μm nylon cloth to collect the cyanobacteria suspension. Cyanobacteria were washed with ASW three times by centrifugation (2000 rpm, 4°C, 6 min) and re-suspended in ASW to isolate them to a concentration of 10^6^−10^7^ cells ml^-1^.

For free amino acid (FAA) analysis, 500 μl of freshly isolated cyanobacteria were first mixed with an equal amount of ultrapure water, sonicated in ice for 3 min, and centrifuged at 15,000 rpm at 4°C for 10 min to collect the cellular extract. Extracts were precipitated in 70% ethanol to remove proteins before being subjected to amino acid analysis as described by Wang et al. [29]. Finally, the FAA content was standardized to the protein content in the extract, which was determined using a protein assay kit (Bio-Rad, CA, USA) with bovine serum albumin as the standard.

Each of the 500 μl of freshly isolated cyanobacteria samples was further lyophilized and weighed to determine chlorophyll (Chl *a*, *b*, *c1*+*c2*) content and δ^13^C and δ^15^N values. Chlorophyll in the dried cyanobacteria was extracted with 90% cold acetone and the levels of Chl *a*, *b*, and *c1*+*c2* were determined spectrophotometrically following Jeffery and Haxo [30]. Prior to determining δ^13^C and δ^15^N values, cyanobacteria samples were acidified with 500 μl 1.0 N HCl and shaken overnight to remove carbonate. Acidified samples (1–2 mg) were combusted in an elemental analyzer (Flash EA-1100 NC, Thermo Finnigan, Waltham, MA, USA) to produce CO_2_ and N_2_ for separately determining isotopic compositions [31]. Isotope ratios (δX) were expressed as the difference in parts per thousand (‰) from standard reference material as indicated below:

δX=((Rsample/Rstandard)−1)×1000;

where X is ^13^C or ^15^N, R is the corresponding ratio of ^13^C:^12^C or ^15^N:^14^N, and δ is a measure of heavy to light isotopes in the sample. The international standards of Pee Dee Belemnite (PDB) for carbon and atmospheric N_2_ for nitrogen were used as references.

### Statistical analysis

Comparisons of the differences in oxygen concentration during photosynthesis and respiration, the contents of chlorophyll pigments, amino acid contents, δ^13^C and δ^15^N values between sponge CF, MP and NCF were made using a one-way analysis of variance (ANOVA) followed by Tukey’s honest significant difference (HSD) test for multiple comparisons at a significance level of 0.05.

## Results

### Photosynthesis of *Terpios* cyanobacteria *in situ*

Cyanobacteria photosynthetic performance was significantly different among the three tested parts of *T*. *hoshinota* (Table 1). The mean *F*_*v*_ */F*_*m*_ value at the growth front in contact with living corals (CF, 0.256 ± 0.014) was significantly lower than for the mid-body (MB, 0.300 ± 0.011) and no-contact front (NCF, 0.319 ± 0.013) (Table 1, *F*_(2,54)_ = 6.186, *P* < 0.01). All three tissue locations contained comparable Chl *a* (*F*_(2,21)_ = 0.201, *P* > 0.05) and Chl *c1*+*c2* (*F*_(2,21)_ = 0.370, *P* > 0.05) content, but not Chl *b*. MB and NCF tissues had significantly higher Chl *b* content than CF tissue (*F*_(2,21)_ = 4.970, *P* < 0.05).

**Table 1:** Changes in the maximum quantum yield obtained from *Terpios*- cyanobacteria symbiosis and photosynthetic pigment content of freshly isolated cyanobacteria at the coral contact front, mid-body, and no-contact front.

	*F*_*v*_ */F*_*m*_	Chl *a*	Chl *b*	Chl *c1+c2*
Coral front	0.256 ± 0.014^a^ (19)	394 ± 71^a^ (8)	5 ± 2^a^ (8)	25 ± 4^a^ (8)
Mid-body	0.300 ± 0.011^b^ (19)	435 ± 40^a^ (8)	27 ± 6^b^ (8)	21 ± 3^a^ (8)
No-contact front	0.319 ± 0.013^b^ (19)	444 ± 87^a^ (8)	29 ± 10^b^ (8)	22 ± 4^a^ (8)
	*F*_2,54_ = 6.538, P < 0.01	*F*_2,21_ = 0.201, P > 0.05	*F*_2,21_ = 4.970, P < 0.05	*F*_2,21_ = 0.370, P > 0.05

The content of pigments is standardized to dry weight of sample and expressed as μg g^-1^ dry wt. Data are means ± S.E., followed by the number of replicates in parentheses. Means followed by the same letter are not significantly different at *P* = 0.05 (Tukey’s honest significant difference test).

### Laboratory measurement of photosynthetic capability and dark respiration rate

**Fig 2:**
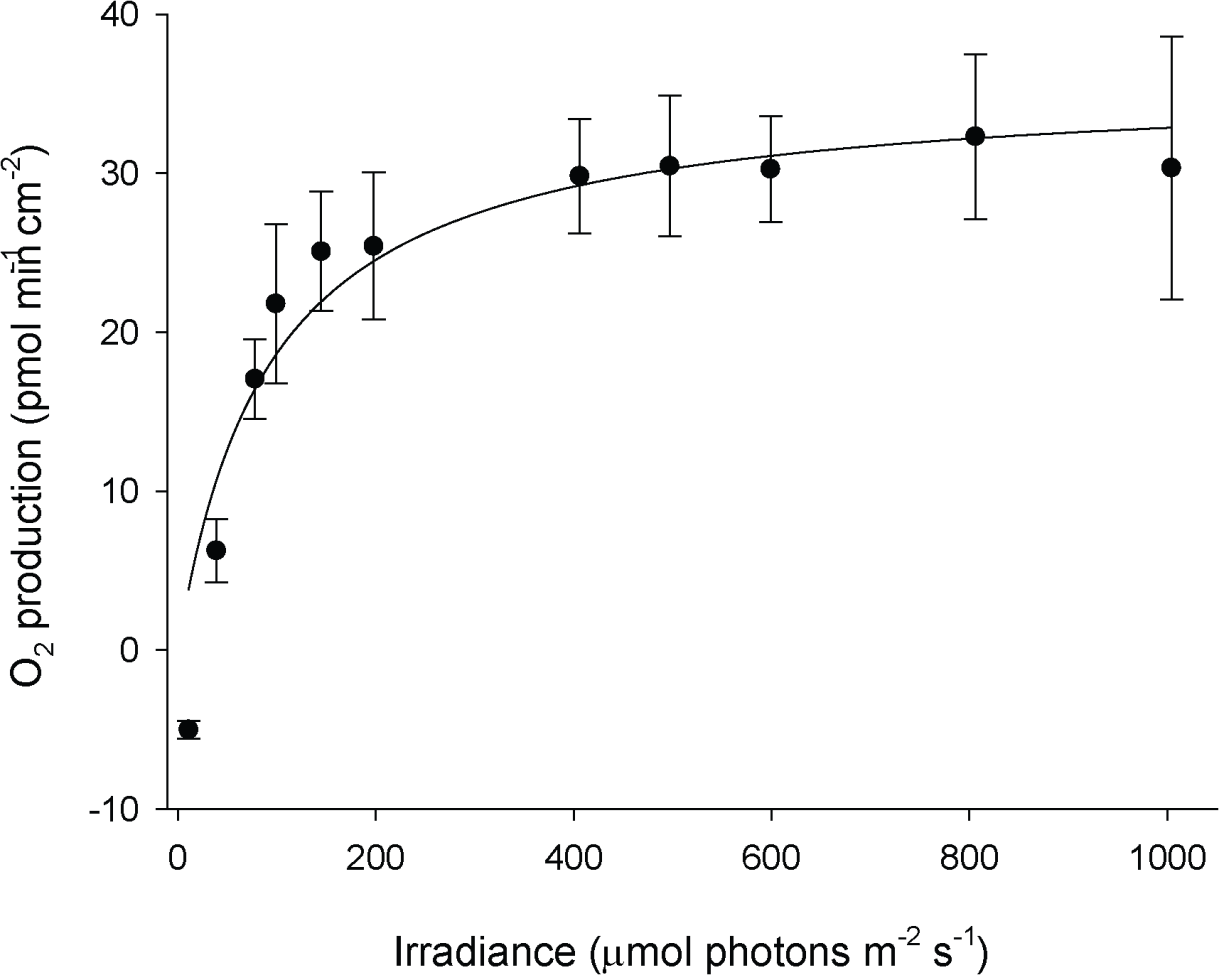
Photosynthesis-irradiation curve of cyanobacteria in *Terpios hoshinota* encrusting on coral skeleton. Data are means ± SD (n = 3).

**Fig 3:**
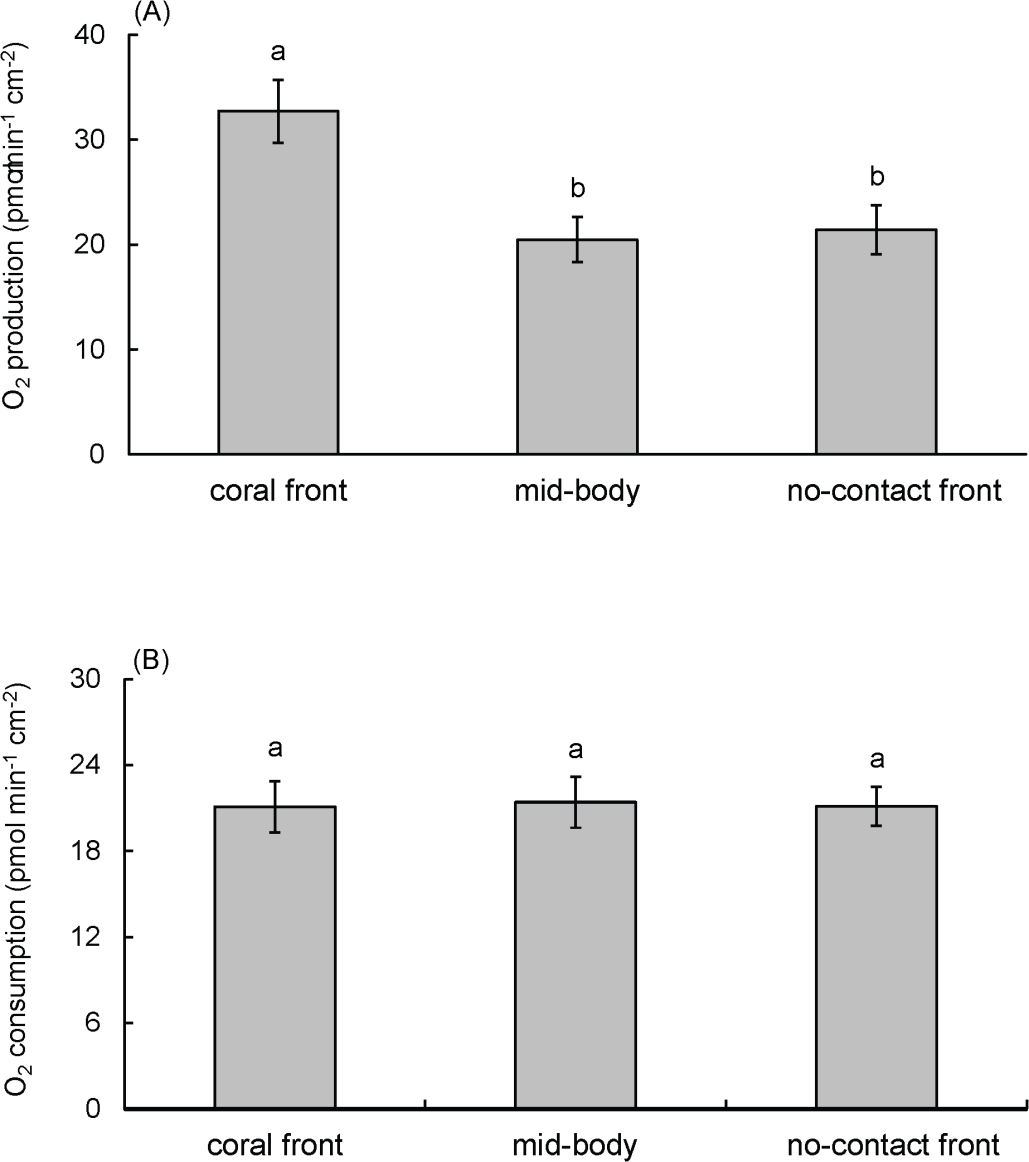
Net photosynthetic oxygen production of cyanobacteria within *Terpios hoshinota* under 400 μmol photons m^-2^s^-1^ (A) and total oxygen consumption of the sponge (B) collected from coral contacting front, mid-body, and no-contact front. **Data are means ± S.E. (n = 17).** Bars with the same letter are not significantly different at *P* = 0.05 (Tukey’s honest significant difference test). The data displayed are the combined results of two separate experiments.

**Fig 4:**
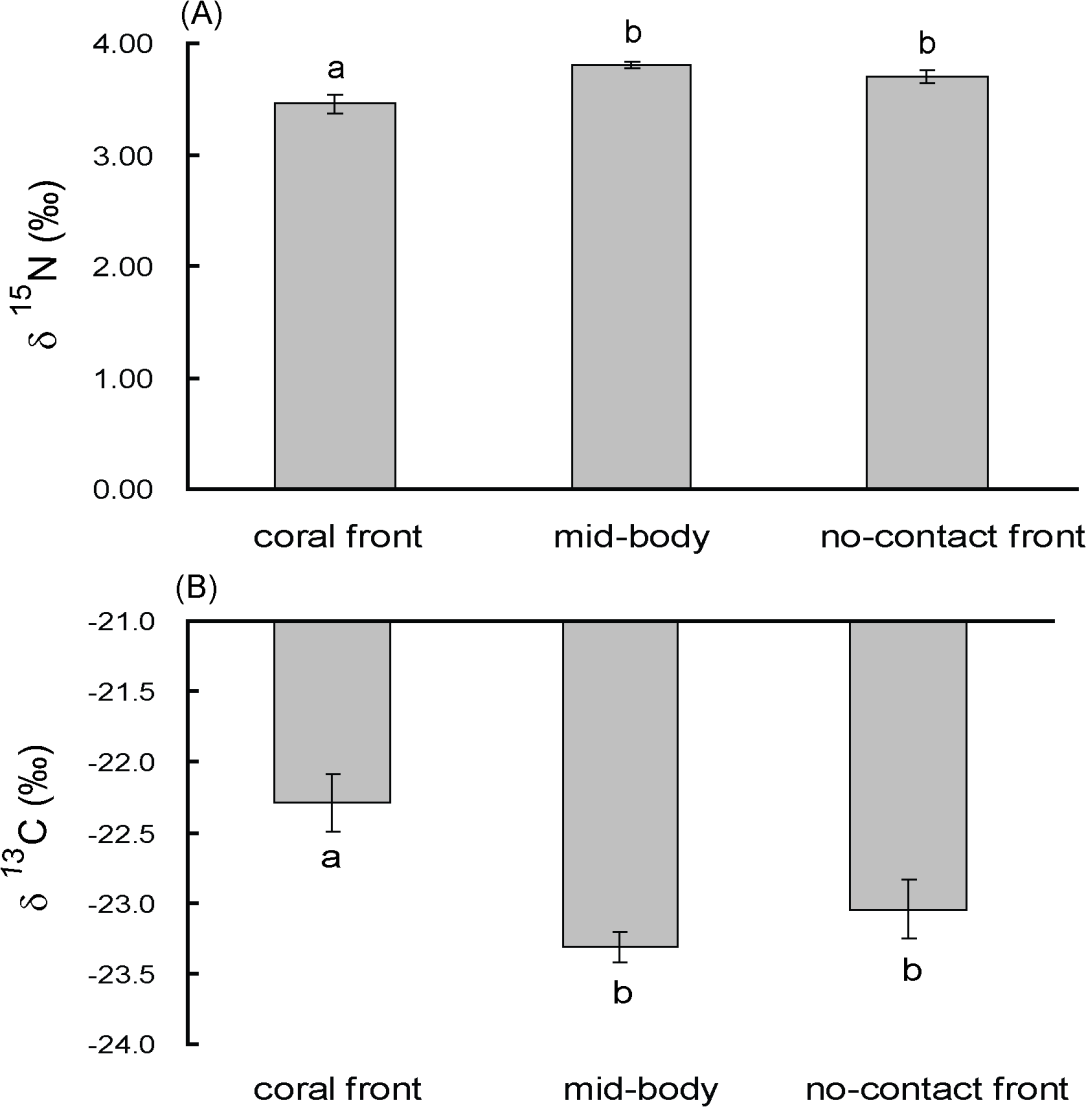
Changes in δ^15^N (A) and δ^13^C (B) values of isolated symbiotic cyanobacteria from *Terpios hoshinota* at the growth front where it contacts the coral, mid-body, and non-contact front. Data are means ± S.E. (*n* = 8). Bars with the same letter are not significantly different at *P* = 0.05 (Tukey’s honest significant difference test).

Physiological differences in the three *Terpios* body locations were detected during photosynthetic oxygen production and dark respiration. The photosynthesis-irradiance (P-I) curve of the sponge CF was determined before examining photosynthetic performance. *Terpios* reached its maximum photosynthetic rate at an irradiance of > 400 μmol photons m^-2^s^-1^ and no photoinhibition occurred below 1,000 μmol photons m^-2^s^-1^ (Fig 2). The data obtained at irradiances >1,000 were not used in this study because they caused respiration chamber temperatures to rise. The variables shown in Fig 2 fitted well into a non-photoinhibition equation model (*P* = *P*_max_ (*I*)/ *KI* + (*I*); r^2^ = 0.910). With this equation, the calculated *P*_max_ (maximum photosynthetic rate) was 36 ± 3 *p*mol O_2_ min^-1^ cm^-2^, and *KI* (half saturation constant) was 93 ± 28 μmol photons m^-2^s^-1^. When comparing photosynthetic performance between *Terpios* from the CF, MB, and NCF (*n* = 17 for each parts) at an irradiance of 400 μmol photons m^-2^s^-1^, sponges displayed significantly higher photosynthetic rates in the CF samples (33 ± 3 *p*mol min^-1^ cm^-2^) than in MB (20 ± 2 *p*mol min^-1^ cm^-2^) and NCF samples (21 ± 2 *p*mol min^-1^ cm^-2^) (*F*_(2,48)_ = 6.538, *P* < 0.01) (Fig 3A). Measured from the same *Terpios* sample, the three different sampled parts had comparable dark respiration rates (Fig 4B), which were 21 ± 2 *p*mol min^-1^ cm^-2^ for CF and MB and 21 ± 1 *p*mol min^-1^ cm^-2^ for NCF (*F*_(2,48)_ = 0.023, *P* > 0.05).

### Physiological index determination in isolated cyanobacteria

To explore the influence of coral contact on the nitrogen status of endosymbiotic cyanobacteria in *Terpios*, total FAA and the Gln/Glu ratio were measured. Total free amino acids (FAA) in freshly isolated cyanobacteria from CF sponge tissue were about 1.9 and 1.7 times higher than from MB and NCF, respectively (Table 2, *F*_(2,21)_ = 11.758, *P* < 0.01). No significant difference in FAA content was found between MB and NCF (*P* > 0.05). Taurine, a non-proteinogenic amino acid, was present as a significant molar percentage (27~35 mol%) of total FAA, but displayed no significant differences (*F*_(2,21)_ = 1.257, *P* > 0.05) among the different parts of *Terpios* (Table 2). In contrast, CF cyanobacteria exhibited similar increases in proteinogenic amino acids when compared to MB and NCF (*F*_(2,21)_ = 6.704, *P* < 0.01). FAA analyses also revealed that cyanobacteria contained remarkably higher glutamate (15.0 ± 3.5, 15.5 ± 1.7, and 17.8 ± 2.9 mol% for the CF, MB, and NCF, respectively) than glutamine (0.7 ± 0.1, 0.6 ± 0.1, and 0.4 ± 0.1 mol% for the CF, MB, and NCF, respectively), resulting in very low Gln/Glu ratios (Table 2). However, the Gln/Glu ratios from the CF (0.14 ± 0.02) were still about 2 times higher than from the MB (0.06 ± 0.02) and NCF (0.07 ± 0.03) (*F*_(2,21)_ = 6.144, *P* < 0.01).

**Table 2:** Free amino acids in *Terpios hoshinota* cyanobacteria collected from the coral contact front, mid-body, and no-contact front.

	Total	Proteinogenic	Taurine (mol%)	Gln/Glu
Coral front	2,273 ± 312^a^	1,615 ± 314^a^	31 ± 5^a^	0.14 ± 0.02^a^
Mid-body	1,193 ± 104^b^	767 ± 101^b^	35 ± 3^a^	0.06 ± 0.02^b^
No-contact front	1,346 ± 91^b^	981 ± 84^b^	27 ± 4^a^	0.07 ± 0.03^b^
	*F*_2,21_ = 11.758, P < 0.01	*F*_2,21_ = 6.704, P < 0.01	*F*_2,21_ = 1.257, P > 0.05	*F*_2,21_ = 6.144, P < 0.01

The content of free amino acids is standardized to soluble protein content of sample and expressed as pmol mg^-1^. Means followed by the same letter are not significantly different at *P* = 0.05 (Tukey’s honest significant difference test). Data are means ± SE (n = 8).

Freshly isolated cyanobacteria from *Terpios* CF, MB, and NCF were measured for their δ^13^C and δ^15^N values. Cyanobacteria (*n* = 8 for each sampling part) isolated from the CF had significantly lower C/N ratios (5.20 ± 0.05) than MB (5.34 ± 0.03) and NCF (5.32 ± 0.04) (*F*_(2,21)_ = 3.844, *P* < 0.01). When comparing stable isotope ratios, cyanobacteria isolated from the CF displayed less nitrogen (3.45 ± 0.09‰) than the MB (3.80 ± 0.03‰) and NCF (3.70 ± 0.17‰); however, the differences were within the detection error of the elemental analyzer used in this study. Conversely, δ^13^C values from CF cyanobacteria (-22.29 ± 0.20‰) were significantly heavier than MB (-23.31 ± 0.11‰) and NCF (-23.05 ± 0.21‰) (*F*_(2,21)_ = 8.239, *P* < 0.01).

## Discussion

*Terpios hoshinota* was first discovered in Guam four decades ago [10], and since then the occurrences of coral overgrowth outbreaks have been continuously reported from the Pacific Ocean and Indian Ocean [7,10–13,15–18]. However, the interactions between *T*. *hoshinota* and opponent corals were not explored until recently, with results suggesting a significant change in *Terpios* morphology when combating corals [23]. By measuring the physiological characteristics of cyanobacteria from different parts of the sponge, our study represents the first to demonstrate that *T*. *hoshinota* competes with opponent corals not only by morphological transformation of the sponge-cyanobacteria association but also by physiological outperformance (*e*.*g*., higher photosynthetic rate and nitrogen status) in order to accumulate resources for fighting with corals.

### Physiological characteristics of cyanobacteria in different parts of *Terpios hoshinota*

As a sponge species containing a high density of photosynthetic cyanobacteria, the physiological performance of *Terpios* is expected to closely link to the photosynthetic activity of the cyanobacteria. To evaluate the photosynthetic performance of the *Terpios*-cyanobacteria association, darkness adapted maximum quantum yield (*F*_*v*_ */F*_*m*_) and oxygen production rate were measured within the three regions of the sponge. Though maximum quantum yield was commonly used to examine the health status of algal symbiotic coral and cyanobacteria [25–27], *F*_*v*_ */F*_*m*_ values of the cyanobacteria symbiotic with *Terpios hoshinota* were relatively low (0.256–0.319) compared to measurements from most corals, which have *F*_*v*_ */F*_*m*_ values of > 0.6 in non-stressed situations. The Campbell *et al*. [32] review of the chlorophyll fluorescence of cyanobacterial photosynthesis concludes that cyanobacteria containing high concentrations of phycocyanin usually show low *F*_*v*_ */F*_*m*_ values. The cyanobacteria association in *T*. *hoshinota* is dominated by an isolated group closely related to *Prochloron* spp. [22], and microscopic anatomy and pigment characterization show that it also contains a high concentration of phycocyanin [19], suggesting that a low *F*_*v*_ */F*_*m*_ ratio is an expected pattern in cyanobacteria associated with *T*. *hoshinota*. In addition, higher maximum quantum yield in the *Terpios* MB and NCF might be attributed to their higher Chl *b* in the photosynthetic pigment assembly of this strain of cyanobacteria (Table 1), because Chl *b* is able to amplify photosystem-II (PSII) functional Chl antenna size about 3.4 times higher than with only Chl *a* [33]. Furthermore, low *F*_*v*_ */F*_*m*_ in healthy cells can be a measurement artefact when the light source does not provide sufficient intensity to saturate PSII [34], and modification to the light source is needed to obtain accurate *F*_*v*_ */F*_*m*_ values [35]. Because the many potential interferences might affect different parts of *Terpios*, *in situ* maximum quantum yield might not be a suitable indicator to determine the photosynthetic performance of *T*. *hoshinota*.

The higher photosynthetic performance in *Terpios* CF was supported by the *ca*. 61% increase in photosynthetic oxygen production that was measured in the laboratory. Comparatively lower photosynthetic performance within *Terpios* MB and NCF were not due to aging or damage of the *Terpios*-cyanobacteria symbiosis because dark respiration rates in the sponge and the contents of major photosynthetic pigment (Chl *a* and *c1+c2*) in the cyanobacteria were comparable among *Terpios*’ three different body parts. In addition, the maximum quantum yield of sponges measured in the field at the MB and NCF were even higher than at the CF, supporting the observation in the laboratory that low photosynthetic performance in the MB and NCF was not due to aging or damage to cyanobacteria.

### Accumulation of resources to fight with opponent corals?

Why does *T*. *hoshinota* from the CF have a significantly higher photosynthetic performance than the MB and NCF? One hypothesis is that the *Terpios* CF needs to accumulate resources including nutrients for cell proliferation to fight opponent corals. Our previous study showed that when encountering coral opponents, *Terpios* develops morphological modifications in preparation for the battle by packing itself with cyanobacteria, sponge tissues, and spicules [23]. This change might require sponge cell and spicule regeneration and particularly high cyanobacteria proliferation resulting in a higher density of germs per unit area of the *Terpios* CF than MB and NCF. The higher performance of these physiological parameters might result from the higher cell density at the *Terpios* CF. This hypothesis is supported by scanning electron microscope imaging in which “hairy” tips at the *Terpios* edge are packed with a high density of cyanobacteria and spicules [22,23]. However, cyanobacteria density in *Terpios* is nearly impossible to determine accurately since *Terpios* tissue penetrates deeply into the undulating coral skeleton matrix [19], making it difficult to recover all cyanobacteria from the coral skeleton substrate. Even with painstaking air/seawater brushing, the coral skeleton substrate still contained blackish cyanobacteria. Nevertheless, a higher proliferation rate of cyanobacteria in the *Terpios* CF was indirectly indicated by higher δ^13^C values than in the MB and NCF (Fig 4A), since a high growth rate would reduce stable carbon isotope fractionation in many marine phytoplankton taxa including cyanobacteria [36,37]. Direct evidence to demonstrate the increase in proliferation rate will be further determined by more precise techniques such as the BrdU staining and cell cycle analysis [38,39].

### Does coral provide a nitrogen source for *Terpios hoshinota*?

The second scenario for explaining the elevation of photosynthesis at the *Terpios* CF might be ammonium enrichment derived from the disintegration of coral tissues overgrown by the sponge. Seawater at the Green Island sampling site is very low in nitrogen (with non-detectable NH_3_, NO_2_^-1^, and NO_3_^-1^ from P-J Meng unpub. data). The oligotrophic environment at Green Island might create ammonium-limiting conditions for symbiotic cyanobacteria in *Terpios* as described for coral symbiosis by Cook et al. [40]. It is also well documented that prolonged exposure to ammonium enrichment by nitrogen-starved phytoplankton elevates algal photosynthetic rates [41–43]. Therefore, abundant ammonium provided by disintegrating coral tissue at the CF might elevate cyanobacteria photosynthesis in the sponge compared to the MB and NCF.

A higher ammonium supply could also explain the slightly higher discrimination of ^15^N in CF cyanobacteria because an increase in dissolved inorganic nitrogen would elevate stable nitrogen isotope fractionation [44–45]. Furthermore, restricted ammonium enrichment at the coral contact front might be attributed to a strong dilution effect in the water column and the limited internal transportation efficiency in a *Terpios* body cavity having a crowded cyanobacteria population. This possibility was also supported by total and proteinogenic FAA contents being higher in CF cyanobacteria than in MB and NCF tissues (Table 2). It has been suggested that internal FAA levels in phytoplankton and photosynthetic cyanobacteria are mediated by the external ammonium supply [46–47].

In conclusion, our data on photosynthetic capability, nitrogen status, and stable carbon isotope data imply that *Terpios* might have to accumulate resources at the sponge edge to fight opponent corals for substrates. Such resources might be derived from photosynthates released by cyanobacteria or supplied by inorganic nitrogen during the process of overgrowing encountered corals, which were higher than in distal parts of sponge tissues not in contact with corals. However, the question of whether *Terpios* kills coral for nutrients or only competes for substrate and obtains nutrients as byproducts is still unanswered. More studies are needed to resolve this puzzle, especially since there are more than four types of boundary interactions between *Terpios* and corals [23] and *Terpios* does not always win the competition.
